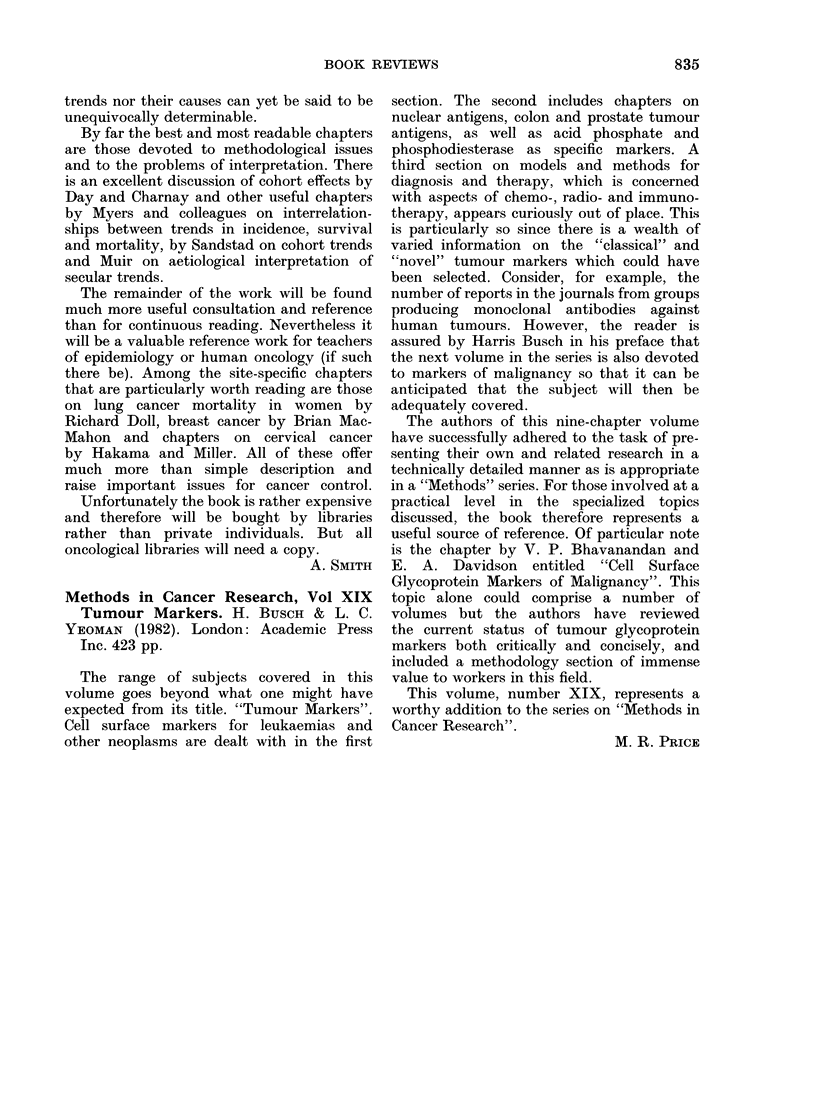# Methods in Cancer Research, Vol XIX Tumour Markers

**Published:** 1982-11

**Authors:** M. R. Price


**Methods in Cancer Research, Vol XIX Tumour Markers.** H. Busch & L. C. Yeoman (1982). London: Academic Press Inc. 423 pp.

The range of subjects covered in this volume goes beyond what one might have expected from its title. "Tumour Markers". Cell surface markers for leukaemias and other neoplasms are dealt with in the first section. The second includes chapters on nuclear antigens, colon and prostate tumour antigens, as well as acid phosphate and phosphodiesterase as specific markers. A third section on models and methods for diagnosis and therapy, which is concerned with aspects of chemo-, radio- and immunotherapy, appears curiously out of place. This is particularly so since there is a wealth of varied information on the "classical" and "novel" tumour markers which could have been selected. Consider, for example, the number of reports in the journals from groups producing monoclonal antibodies against human tumours. However, the reader is assured by Harris Busch in his preface that the next volume in the series is also devoted to markers of malignancy so that it can be anticipated that the subject will then be adequately covered.

The authors of this nine-chapter volume have successfully adhered to the task of presenting their own and related research in a technically detailed manner as is appropriate in a "Methods" series. For those involved at a practical level in the specialized topics discussed, the book therefore represents a useful source of reference. Of particular note is the chapter by V. P. Bhavanandan and E. A. Davidson entitled "Cell Surface Glycoprotein Markers of Malignancy". This topic alone could comprise a number of volumes but the authors have reviewed the current status of tumour glycoprotein markers both critically and concisely, and included a methodology section of immense value to workers in this field.

This volume, number XIX, represents a worthy addition to the series on "Methods in Cancer Research".

M. R. Price